# Fire lines adjacent to aspen are unlikely to hold during extreme burning conditions in southern Rocky Mountain forests

**DOI:** 10.1002/eap.70249

**Published:** 2026-05-08

**Authors:** Trevor A. Carter, Jennifer K. Balch, Maxwell C. Cook, Sarah J. Hart

**Affiliations:** ^1^ Department of Forest and Rangeland Stewardship Colorado State University Fort Collins Colorado USA; ^2^ Environmental Data Science Innovation and Inclusion Lab (ESIIL) University of Colorado Boulder Boulder Colorado USA; ^3^ Colorado Forest Restoration Institute Colorado State University Fort Collins Colorado USA

**Keywords:** fire suppression, quaking aspen, southern Rocky Mountains, western USA, wildland fire

## Abstract

Increases in area of extent, severity, and frequency of wildfires across the western United States are presenting challenges to socio‐ecological systems, including shifts to alternative ecological states, loss of homes, and compromising human health. Wildfire suppression operations, such as constructing hand lines to limit the spread of fire, are an important part of wildland fire management, particularly in the wildland urban interface. Like other attributes of wildland fire activity and effects, suppression strategies and their effectiveness vary with ecological, topographic, climatological, and sociopolitical factors. However, there has been little research that examines the efficacy of suppression operations, specifically as they relate to forest composition. Here, we ask about the effectiveness of fire line construction based on adjacent stand composition. Specifically, we ask: (1) Are wildfire suppression lines preferentially constructed in stands with specific tree species? (2) How does species identity influence the probability that suppression lines hold when also considering differences in topography, climate, and extreme fire weather? We anticipated that suppression operations will be biased towards—and more effective in—stands with quaking aspen because they are often associated with less extreme fire behavior than many conifer species. We conducted our study in the southern Rocky Mountain ecoregion using fires (*n* = 36) that burned during 2019–2023 and included records of fire suppression operations (*n* = 4295). We used nonparametric statistical models to elucidate biases in the construction of fire lines and the effects of stand composition. We found quaking aspen was the least common tree species to be within fire footprints, yet fire lines were placed near quaking aspen 1.68–5.30 times more than commonly co‐occurring tree species. Fire growth, independent of stand composition, was the most important predictor for whether fire suppression lines were likely to hold but the percentage of fire lines that held differed between fires >40,500 ha and smaller events (65% vs. 82%, respectively). This research suggests that wildland firefighters preferentially located fire lines near aspen stands, perhaps due to the long‐held notion that aspen stands are less flammable. However, during extreme burning conditions fire lines are unlikely to hold regardless of stand composition.

## INTRODUCTION

Wildfire (hereafter fire) in the western United States is increasing in area of extent, severity, frequency, and growth rate (Balch et al., 2024; Chandler et al., 2024)—leading to consequences for both human structures and health (Gould et al., 2024), and the ecology of forested landscapes (Coop et al., 2020; McFarland et al., 2025). Recent changes in fire activity and effects across the western United States are largely explained by a combination of a legacy of fire suppression (Akhtar, 2020) and extreme atmospheric moisture deficits (Higuera & Abatzoglou, 2021), which interact and result in anomalous fire conditions (Kreider et al., 2024). Extreme atmospheric moisture deficits dry out fuels, increasing the likelihood of ignition and the potential for rapid fire spread, particularly in systems where fuels are not limiting (Marlon et al., 2012). Across the western United States, variability in vapor pressure deficit explained 56% of the variability of burned area from 1984 to 2020 (Higuera & Abatzoglou, 2021). The combination of fire exclusion and suppression through the cessation of indigenous burning, grazing, and timber harvest has led to increases in fuels, particularly in forests characterized by frequent, low‐severity wildfire prior to Euro‐American colonization (Boisramé et al., 2022; Collins et al., 2011; Parks et al., 2018; Schoennagel et al., 2004). Collectively, extreme weather conditions and historic fire suppression have created conditions that challenge fire suppression efforts (Kreider et al., 2024).

Despite these challenges, there is a cultural expectation to suppress fires in the western United States (Raish et al., 2007), especially when there is a potential impact on human structures or lives. Suppression efforts can vary locally in method (e.g., constructing hand lines, dozer lines) based on surrounding environmental conditions and management goals (e.g., decrease soil erosion; Backer et al., 2004). Thus, it is important to understand the efficacy of suppression operations across a variety of forest and landscape conditions, both in the context of extreme fire weather which promotes fast‐growing, difficult to suppress fires (Balch et al., 2024), and under less extreme fire weather when fires are more easily managed (Kreider et al., 2024). In addition to atmospheric moisture deficits, topography (e.g., elevation, slope, topographic position) and fuels (e.g., canopy cover, canopy bulk density) can impact the spread and behavior of fires (Povak et al., 2018). Fuel complexes often differ predictably between deciduous (e.g., quaking aspen; *Populus tremuloides*) and coniferous (e.g., pine, spruce, and/or fir; *Pinus* spp., *Picea* spp., *Abies* spp.) forests leading to differences in fire behavior and effects (DeRose & Leffler, 2014; Nesbit et al., 2023), with deciduous forests being linked to decreased fire spread and intensity in Canadian boreal forests (Hély et al., 2000). In the western United States, there is increasing interest in lowering fire risk near the wildland–urban interface through the conversion of highly flammable conifer forests to forests composed of tree species associated with decreased fire activity, such as quaking aspen (*P. tremuloides*). However, there is conflicting evidence on the role of aspen in modifying fire activity, which warrants further exploration.

Quaking aspen is a widespread deciduous species that has been associated with decreased fire severity, occurrence, and behavior when dominant in the overstory (Harris et al., 2025; Nesbit et al., 2023; Shinneman et al., 2013). However, these effects are not unanimous (Nesbit et al., 2023) likely due to the variety of moisture conditions (LaMalfa & Ryle, 2008), fuel loads (Hély et al., 2000), and codominance with conifer species (Bigler et al., 2005; Dickinson & Johnson, 2004). Extreme fire weather also plays an important role in driving fire occurrence and spread in aspen‐dominated forests. For example, in moderate weather conditions, fire spread in aspen may be less extreme (Dash et al., 2016), but under more extreme weather conditions surface fires may behave similar to conifer forests, with increased likelihood of active crown fires (DeRose & Leffler, 2014). While previous studies have documented the effect of aspen forests on fire occurrence, behavior, and severity (Harris et al., 2025, Nesbit et al., 2023), the role quaking aspen plays in the effectiveness of fire suppression operations remains unknown. There is a critical need to determine if there are natural landscape or vegetation features that aid in suppression effectiveness.

Here, we evaluate the distribution of fire suppression operations, namely fire lines (i.e., hand lines, dozer lines, etc.), in fires across the southern Rocky Mountains and their effectiveness based on stand composition. Specifically, we ask: (1) Are wildfire suppression lines preferentially constructed in stands with specific tree species? (2) How does species identity influence the probability that suppression lines hold when also considering differences in topography, climate, and extreme fire weather? Given the anecdotal information of the effectiveness of aspen in wildland fire fighting, and the association of aspen stands with less extreme fire behavior relative to neighboring conifer species (Nesbit et al., 2023), we anticipate that suppression operations will be biased towards—and more effective in—stands dominated by quaking aspen under non‐extreme fire weather conditions. We predict this will translate into increased efficacy of suppression operations, defined by the success of fire line engagement, under non‐extreme conditions. However, under extreme conditions, we expect stand composition will not influence the success of suppression operations, rather fire weather will be the primary predictor of the success of fire suppression operations.

## METHODS

### Study area

**FIGURE 1:**
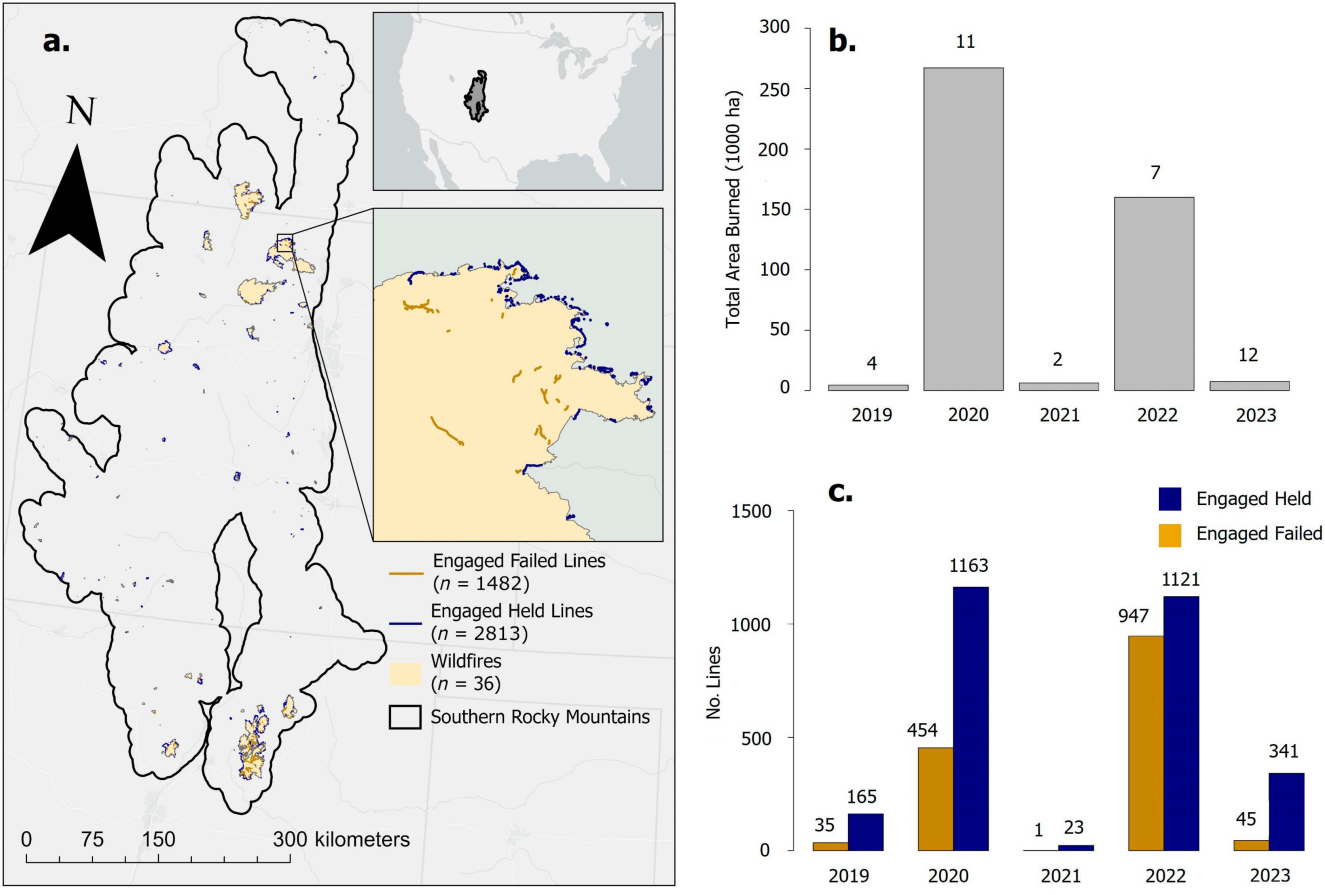
(a) Map of the southern Rocky Mountain ecoregion. Green polygons represent wildfires from 2019 to 2023 with fire lines (*n* = 36). Gold lines represent engaged fire lines that burned over (failed), blue lines represent engaged fire lines that did not burn over (held). (b) Distribution of total area burned from 2019 to 2023. The number above the bars represents the number of fires counted in the total fire year for that year. (c) Number of engaged held (blue) and engaged failed (gold) for each year. The number above the bars represents the number of lines for each category.

The southern Rocky Mountain ecoregion covers approximately 15 M ha, over 7 M ha of which are forested (Figure 1). The region spans from southern Wyoming, through Colorado, into New Mexico, with northern latitudes experiencing wetter and cooler climates relative to the southern latitudes (Peet, 1978, 2000). Elevation across the region varies from approximately 1350 to 4400 m above sea level. The composition of forests in the southern Rocky Mountains varies across latitudinal and elevational gradients, with the foothills and drier parts of the ecoregion composed of shortgrass prairie, xeric shrublands, and Rocky Mountain juniper (*Juniperus scopulorum*)/pinyon pine (*Pinus edulis*) woodlands. As water becomes less limiting, ponderosa pine (*P. ponderosa*) and Gambel oak (*Quercus gambelii*) are dominant at lower elevations, while montane mixed‐conifer forests of ponderosa pine, white fir (*Abies concolor*) and Douglas‐fir (*Pseudotsuga menziesii*) are prevalent at higher elevations. In more southern latitudes, lower subalpine forests are dominated by Douglas‐fir and subalpine fir (*Abies lasiocarpa*), while more northern latitudes are composed of Douglas‐fir and lodgepole pine (*P. contorta*). Upper subalpine forests across the southern Rocky Mountain ecoregion are dominated by Engelmann spruce (*Picea engelmannii*) and subalpine fir in mesic areas, and limber pine (*P. flexilis*) in more xeric areas (Peet, 1978). Notably, quaking aspen is present across both latitudinal and elevational gradients, spanning from mesic ravine bottoms to xeric slopes (Peet, 1978), and comprising about 10% of forested area in the southern Rocky Mountains (Cook et al., 2024).

### Operations data

To understand how the effectiveness of fire suppression operations varies across this topographically and ecologically complex system, we obtained operations data for recent wildfires (2019–2023) from the National Interagency Fire Center (Figure 1b; Table 1). This timeframe aligns with recent high resolution remotely sensed aspen canopy maps in the region (see *Environmental and vegetative data*). Operational data archive contained all managed incidents (fire perimeters) for the United States as well as the fire suppression lines. We extracted fire perimeters that were within 10 km of the southern Rocky Mountains. Each fire perimeter was manually assessed to ensure geometries were valid and that obvious errors were removed (e.g., sharp angles in isolated areas or straight lines that spanned long distances where fire lines could not reasonably be placed) using ArcGIS Pro (version 4.2.1) and the terra package in R (Hijmans et al., 2025). Of the fires included, only four burned areas were greater than 40 k ha: Cameron Peak, East Troublesome, Hermit's Peak, and Mullen (Table 1). These four fires alone accounted for over 80% of the total burned area in our study region during this time period.

**TABLE 1:** Details on the fire events (*n* = 36) including fire name, year, area burned (in hectares), the number of held and failed fire lines, and the average fire line length (in meters) associated with each event.

Fire name	Year	Area (ha)	Held lines	Failed lines	Avg. line length (m)
403	2023	615	101	13	64
Black Feather	2023	952	7	16	615
Calwood	2020	4100	17	2	845
**Cameron Peak**	2020	84,500	492	96	252
Cerro Pelado	2022	18,500	47	25	514
Chris Mountain	2023	207	13	2	594
Coalmine	2023	118	13	4	300
Cow Creek	2019	348	1	0	308
Decker	2019	3600	155	31	616
East Canyon	2020	1200	54	1	483
**East Troublesome**	2020	78,000	106	59	402
El Valle	2023	213	15	0	464
Grizzly Creek	2020	13,000	187	26	361
**Hermit's Peak**	2022	138,000	831	816	432
High Park	2022	636	12	5	393
Hope	2023	522	1	0	304
Lefthand	2020	190	25	3	435
Little Mesa	2023	1620	0	1	577
Lowline	2023	769	52	4	537
Middle Fork	2020	8270	15	4	656
Middle Mamm	2019	493	8	4	724
Midnight	2022	1990	180	88	361
Monday Creek	2022	264	6	1	434
Morgan Creek	2021	3070	20	1	405
**Mullen**	2020	71,600	188	253	276
Plumtaw	2022	294	41	11	301
Reveille	2019	73	1	0	42
Saint Charles	2023	199	70	0	283
Simms	2022	150	4	1	370
Spring Creek	2023	1330	53	3	533
Sylvan	2021	1540	3	0	1307
Thorpe	2020	63	6	0	376
Titan	2023	377	9	2	650
Trail Springs	2023	527	7	0	494
Williams Fork	2020	6000	71	10	380
YMCA	2020	123	2	0	889

*Note*: The distribution of line lengths by fire is presented in Appendix S1: Figure S1. Fire names in bold represent large fire events (*n* = 4) that were excluded in secondary analyses.

Next, we extracted engaged fire lines (i.e., those which likely experienced fire) from the operational data archive by identifying fire lines that were within 60 m of the fire perimeters (Gannon et al., 2020). Engaged lines were classified as either engaged and held or engaged and failed, depending on their distance to the fire perimeter. Held lines were within 60 m of the perimeter, whereas failed lines were further than 60 m away but within the interior of the fire (Gannon et al., 2020). We recorded the date of engagement for each of the lines and removed duplicates, such as those whose construction spanned multiple days but covered the same area. It is important to note that fire perimeters may not be completely surrounded by fire lines due to a variety of factors such as aerial tanker drops (outside the scope of this study), fires naturally stopping in the face of topographic features (e.g., rivers), or fires naturally burning out due to changes in fire weather (e.g., late season fires).

### Environmental and vegetative data

We used a digital elevation model (US Geological Survey National Map; 30 × 30 m resolution) to calculate elevation (in meters), derive the slope (in degrees), and topographic position index (TPI). For each month of the 2019–2023 fire seasons, we calculated the departure from 30‐year atmospheric moisture deficits (VPd) using the worldclim dataset (Fick & Hijmans, 2017; 1000 × 1000 m) for the southern Rocky Mountains. We paired our topography (elevation, slope, and TPI) and climatic (departure from 30‐year norm VPd) data with a high‐resolution remotely sensed map of ca. 2019 aspen canopy cover (Cook et al., 2024; 10 × 10 m) and a tree level model of stand composition for the contiguous USA (Riley et al., 2021; 30 × 30 m). We only included tree species that aligned with Peet (2000). We resampled the stand composition map from Riley et al. (2021) to the resolution of the remotely sensed map of aspen using nearest neighbor interpolation (10 × 10 m). Similarly, we resampled all topographic and climatic layers to the resolution of the remotely sensed map of aspen using bilinear interpolation. Finally, we tested for correlation of predictor variables within the data and found no strong (≥|0.7|) correlations within predictor variables (Appendix S1: Figure S2).

### Data processing

We imported the fire lines into R (v4.4.1; R Core Team, 2024) using the terra package (Hijmans et al., 2025) and assigned environmental conditions to each line. Specifically, we extracted the average pixel value for elevation, slope, TPI, and difference in vapor pressure deficit that intersect each line, as well as the proportion of tree species using the dominant forest types from Peet (2000) and data from Cook et al. (2024; aspen) and Riley et al. (2021; other tree species) found along each line. Finally, we used the ICS‐209‐PLUS situation reports, which provide daily snapshots of incident development and response (St. Denis et al., 2023), to extract the daily fire growth (in hectares) for each event. We then assigned fire growth for the date that the fire interacted with each line, as fire growth is an important predictor in determining whether or not a fire line succeeds or fails (Balch et al., 2024). Further, fire growth serves as proxy for fire weather because there are challenges with estimating fire weather at high resolution for any given fire. Our final sample included 4295 fire lines (1482 failed lines, 2813 held lines) spanning 36 fires (Table 1; Figure 1c).

### Prevalence of fire lines adjacent to aspen

To assess the prevalence of fires by vegetation type and the corresponding frequency of constructed fire lines, we counted the total number of pixels for each vegetation type within the study area (including a 10‐km buffer), the total number of pixels by vegetation type contained within burned areas (including a 60 m buffer from the fire perimeter), and the number of pixels by vegetation type touching fire lines (both held and failed). We then used a χ^2^ test of independence to assess whether fires were more likely to occur within certain vegetation types than would be expected by chance and if fire lines within a burn perimeter were more likely to be constructed next to certain vegetation types. Next, we used pairwise comparisons of vegetation types using a Fisher's exact test to quantify the odds of vegetation types burning relative to aspen and the odds of a fire line being constructed in other vegetation types compared to aspen.

### Probability of suppression operation success in aspen forests

To understand the effect of aspen on the capacity for fire lines to hold, we constructed two random forest models. Random forest models are useful machine learning tools capable of explaining nonlinear patterns that are generally robust to overfitting and can account for spatial autocorrelation (Cutler et al., 2007; Povak et al., 2020). As predictors in our models, we included fire growth on date of fire line engagement, fire line length, elevation, slope, TPI, difference in vapor pressure deficit from 30 year normal, and proportion of major forest types from Cook et al. (2024) and Riley et al. (2021) to predict the status of fire lines (held vs. failed). In the first model, we used all data (number of fires = 36; held lines = 2813; failed lines = 1482; Table 1). The second model was constructed excluding data from the four largest fire events, which were all >40,500 ha (and accounted for >80% of total burned area in the study area during the time period), which likely contain extreme growth signals that obfuscate the effects of other variables under more standard conditions (number of fires = 32; held lines = 1196; failed lines = 258; Table 1).

Spatial autocorrelation was highly prevalent in our data because fire lines are not constructed randomly, but rather in distinct spatial clusters. Prior to constructing models, we subsampled the data to include only one observation within a 100 × 100 m cell and thus reduce initial spatial autocorrelation. To further account for spatial autocorrelation in the dataset, we calculated spatial eigenvectors using the principal coordinates of neighbor matrices (PCNM) method (Borcard & Legendre, 2002; Dray et al., 2006). The PCNM method relies on a Euclidean distance matrix truncated to a specified distance determined through a spatial correlogram. The truncated distance matrix is then input into a principal coordinates analysis which returns eigenvectors that represent regions of spatially correlated values. Using these eigenvectors as predictor variables allows models to capture non‐stationarity in predictor–response relationships (Dormann et al., 2007) and reduce spatial autocorrelation in the residuals.

Models were constructed using a spatial cross‐validation approach, where 100 random forest models were independently constructed from a training dataset consisting of a spatial cluster of data points representing 80% of the dataset. The models were then tested on the remaining 20% of the data. For each spatial cluster, the random forest used five blocks to cross validate predictive power based on the specified fraction of the testing data. We used default hyperparameters for the random forest models (*k* = 5; ntree = 500). We balanced the response variable (held vs. failed lines) in the spatial block training data for both the models that include all fires and the models that exclude large fire events (balanced to have equal proportion of held vs. failed lines). We assessed the predictor variable importance through a mean decrease in accuracy averaged across all model runs. Random forest model performance was evaluated using the five spatial blocks to determine the area under the receiver operating characteristic (ROC) curve, out of bag error (Appendix S1: Figure S3), and nonspatial residuals (Appendix S1: Figure S4). Further, to help visualize the partial effects of predictor variables on fire line status, we constructed linear regression models for each random forest model run using the predictor variables in the testing datasets and the predicted line status from the random forest model.

### Relationship between fire growth and stand composition

To further understand potential trends between species identity and fire growth on the effectiveness of wildfire suppression, we conducted paired *t*‐tests to compare the fire growth associated with held and failed lines. We performed separate tests for each forest community type and for both the subset of the data that exclude large fire events and the data that includes all 36 fire events from 2019 to 2023.

## RESULTS

Of the 14.4 M ha within the study area (southern Rocky Mountains and surrounding areas), 444,000 ha burned between 2019 and 2023, 372,000 ha (84%) of which were from the four largest fires. When considering only fires <40,500 ha (100,000 acres), only 258 of the 1454 fire lines across 32 fire events fail (18% failure rate; Table 1). When we consider all 36 fires, the area burned increases from 71,000 to 444,000 ha, and 1482 of the 4295 fire lines fail, resulting in a 35% failure rate.

### Prevalence of fire lines adjacent to Aspen

**FIGURE 2:**
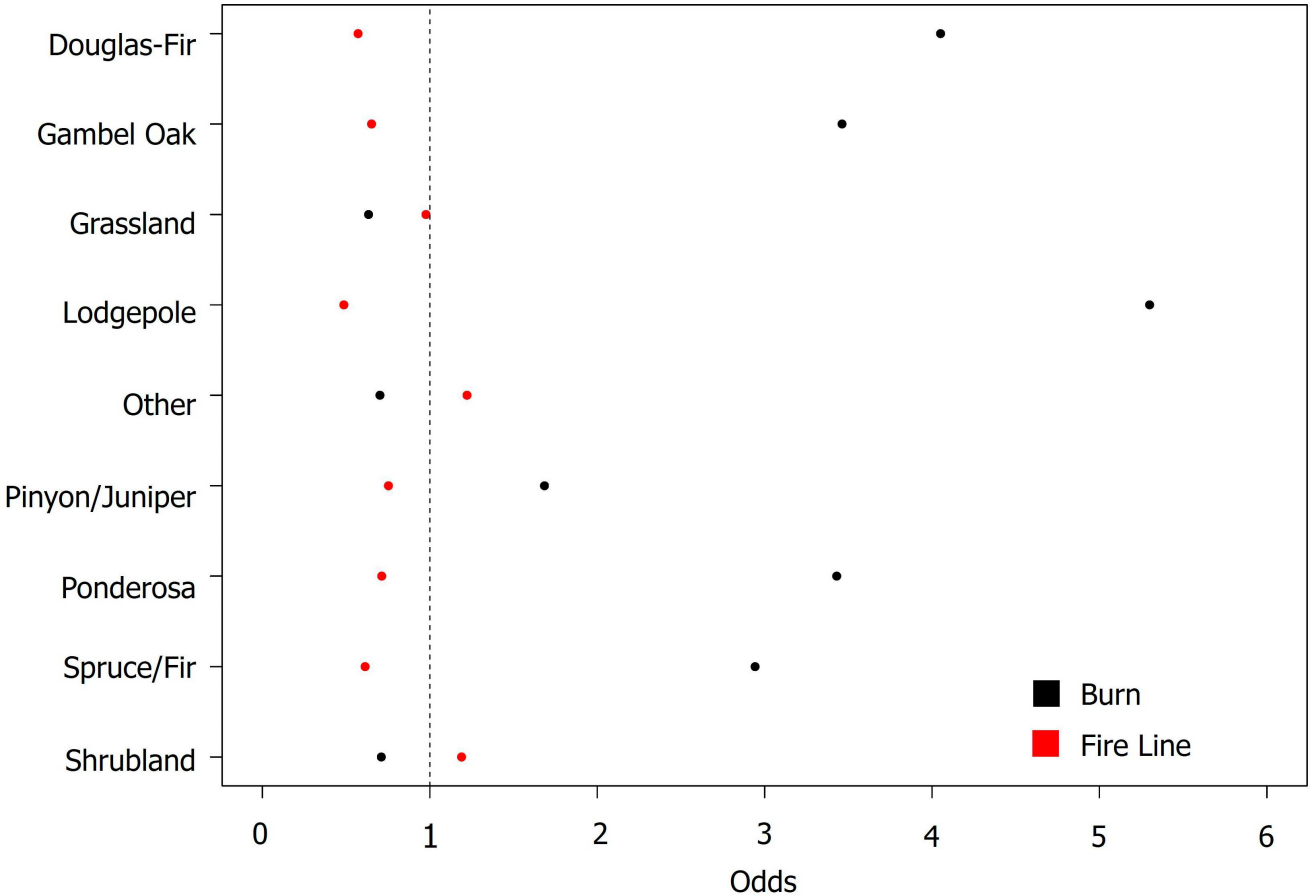
The odds ratios of whether land cover types were more or less associated with burned area (black points) as compared to aspen dominant stands (vertical line at 1) and the odds ratio of whether land cover types were more or less associated with fire lines (red points) as compared to aspen dominant stands (vertical line at 1). A summary of the odds ratios is also presented in Appendix S1: Table S1.

Forests comprised of conifer species that often co‐occur with aspen (e.g., spruce/fir, lodgepole, Douglas‐fir, ponderosa) are the dominant forest types in the southern Rockies (40% of landscape) and were between 2.94 (95% CI: 2.94–2.95), 5.30 (95% CI: 5.29–5.31), 4.05 (95% CI: 4.04–4.06), and 3.43 (95% CI: 3.42–3.44) times (respectively) more likely to burn than aspen forests, which comprise 7% of the landscape (Figure 2; Appendix S1: Tables S1 and S2). In addition, within burned areas, fire lines were 0.613 (95% CI: 0.604–0.623), 0.486 (95% CI: 0.479–0.494), 0.572 (95% CI: 0.563–0.580), and 0.713 (95% CI: 0.702–0.724) times as likely to be constructed next to aspen than spruce/fir, lodgepole, Douglas‐fir, and ponderosa, respectively (Figure 2; Appendix S1: Table S1). In contrast, shrublands, grasslands, and “other” community types (primarily human development) were 0.710 (95% CI: 0.709–0.712), 0.634 (95% CI: 0.633–0.635), and 0.702 (95% CI: 0.701–0.704) as likely to burn compared to quaking aspen (respectively). Similarly, fire lines were 1.19 (95% CI: 1.17–1.21) times more likely to be constructed next shrublands compared to aspen and 1.22 (95% CI: 1.20–1.24) times more likely to be constructed next to “other” community types than aspen (Figure 2), but only 0.977 (95% CI: 0.960–0.994) as likely to be constructed next to grasslands (compared to aspen).

### Probability of suppression operation success and adjacency to aspen forests

**FIGURE 3:**
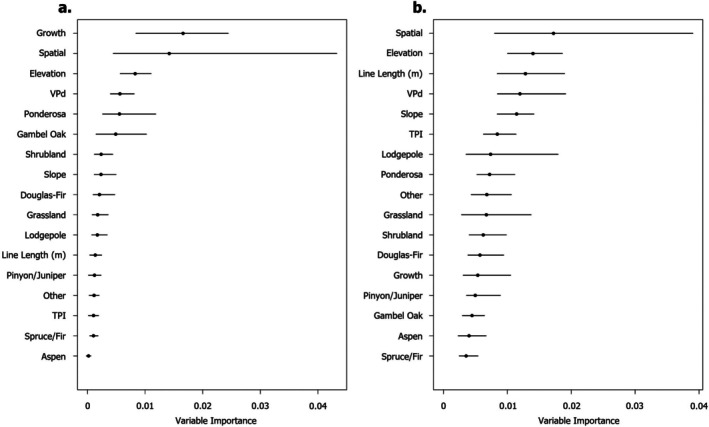
Variable importance plot of the predictor variables included in the random forest model that included all fire events (a) and excluding large fire events (b). The spatial term represents the combined effect of all spatial eigenvectors included to account for spatial autocorrelation. Black points represent the average variable importance from 100 model runs, line segments are the minimum and maximum variable importance for individual variables from any of the 100 model runs. Variables are ordered in decreasing importance based on their average variable importance across model runs.

In the models that include all fire events, fire growth was on average the most important variable with the highest average mean decrease in accuracy (Figure 3a). The spatial eigenvectors (which were included in the model to capture non‐stationarity in predictor–response relationships and reduce spatial autocorrelation in the residuals) had the next highest average mean decrease in accuracy and the widest range of values based on model run (Figure 3a). Elevation and the difference in vapor pressure deficit from 30‐year normal had similar mean decreases in accuracy that were mostly higher than stand composition variables (main exceptions being the proportion of ponderosa and proportion of Gambel oak), line length, slope, and TPI. The proportion of aspen consistently had the lowest mean decrease in accuracy (Figure 3a). In the models that excluded large fire events, the range in mean decrease in accuracy values was greater between model runs. The spatial eigenvectors had the highest average mean decrease in accuracy and a large range (Figure 3b). The remaining variables all had similar mean decreases in accuracy with similarly wide ranges (Figure 3b). On average our random forest models that included all fires predicted the status of fire lines with a 15.1% error rate (Appendix S1: Figure S3a) and a mean area under the ROC curve of 0.925 (range from 0.911 to 0.926 across 100 model runs). Similarly, the random forest models that exclude large fire events predicted the status of fire lines with a 3.20% error rate (Appendix S1: Figure S3b) and a mean area under the ROC curve of 0.996 (range from 0.993 to 0.998 across 100 model runs).

**FIGURE 4:**
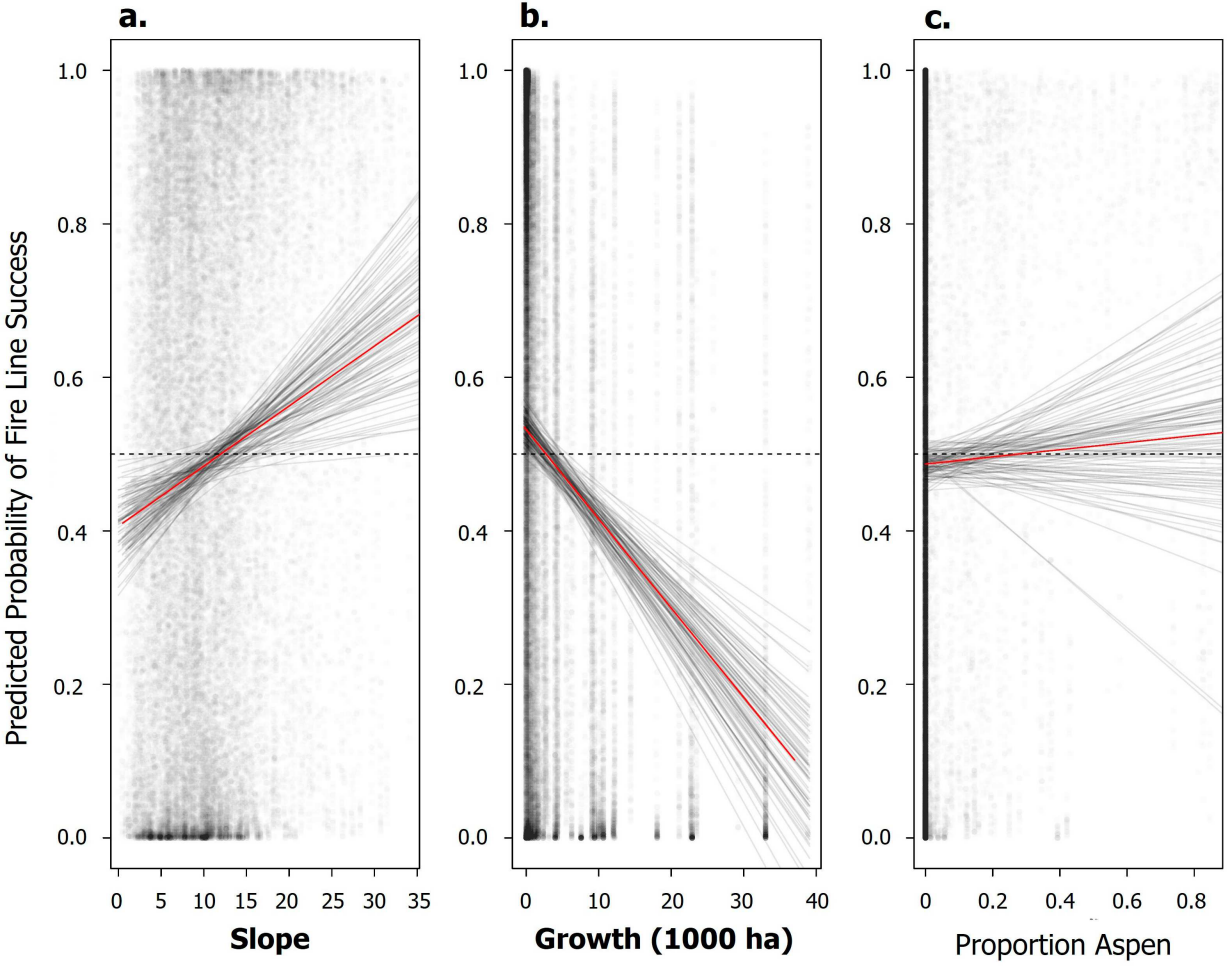
Partial effects of slope (a), fire growth (b), and proportion of aspen (c) from the testing data on the predicted probability of fire lines holding (1 = predicted to succeed, 0 = predicted to fail). Each solid black line represents a single regression using the testing data and predicted line status from 1 of 100 random forest model runs. Points represent the distribution of the data. The red line represents the average partial effect across 100 model runs. Dashed lines represent equal predicted probability of lines holding or failing. Panels with bold *x*‐axis labels indicate which partial effects consistently correlate across all 100 model runs (i.e., all effects positive, or all effects negative). These random forest models and subsequent regression models excluded the four large fire events. These models explained on average 23.6% of the variation in predicted line status.

Only slope and fire growth were consistently correlated (consistent direction of effect across *n* = 100 model runs) with the predicted probability of fire lines holding (Figure 4). Notably, the proportion of quaking aspen was not correlated with the predicted probability of fire lines holding (Figure 4), nor were any other land cover types (Appendix S1: Figure S5). Fire growth was consistently negatively correlated with predicted line status (Figure 4), while slope was positively correlated with predicted line status. Fire lines were predicted to fail when fire growth exceeded 3600 ha and on slopes below 12°. When excluding large fire events, only the proportion of grassland was consistently, and negatively, correlated with predicted fire line status (Appendix S1: Figure S6). We observed no other consistent correlations between predictors and predicted fire line status.

**FIGURE 5:**
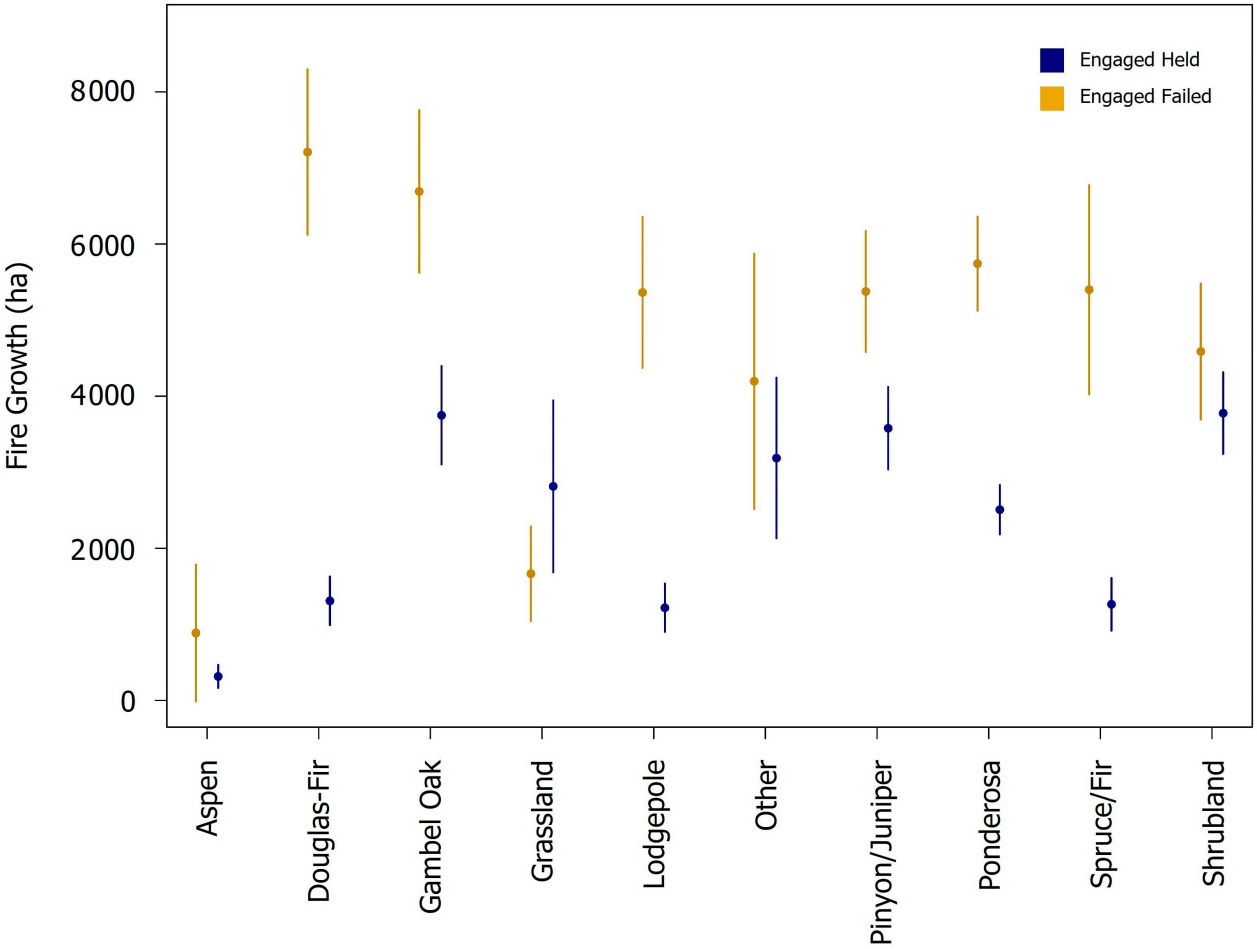
Fire growth across all fire events associated with held (blue points and segments) and failed (yellow points and segments) fire lines based on stand composition. Points represent the average fire growth associated with each category (e.g., held lines in spruce/fir forests). Segments represent the 95% CI around the mean using the formula for 95% CIs assuming a Gaussian distribution.

In general, and when considering all fire events, larger fire growth was associated with the failure of fire lines (Appendix S1: Figure S7); however, the amount of fire growth that corresponded to failed lines varied by land cover type (Figure 5). Interestingly, fire lines in aspen stands, grasslands, “other,” or shrublands had similar fire growth for failed and held lines alike (i.e., not statistically different, *p* > 0.05; Figure 5). Regardless of whether they held or failed, fire lines constructed next to aspen forests were engaged during lower fire growth relative to other land cover types (Figure 5). When only considering non‐large fire events, we observed much less variability in fire growth between held and failed lines across land cover types (Appendix S1: Figure S8). Under less extreme conditions, fire lines in aspen, Douglas‐fir, Gambel oak, lodgepole, pinyon/juniper, ponderosa, and spruce/fir had similar fire growth for failed and held lines (*p* > 0.05). In contrast, shrublands, grasslands, and the “other” category had significant differences in fire growth between held and failed lines under less extreme conditions (*p* < 0.05).

## DISCUSSION

### Prevalence of fire lines

Aspen forests were the least likely forest type to burn; yet more lines were constructed next to aspen forests than any other tree species in the southern Rocky Mountains. This is interesting in part because failed fire lines adjacent to aspen forests were associated with the lowest amount of fire growth. Pure aspen stands have been observed to have decreased fire severity compared to conifer‐dominated stands (Nesbit et al., 2023; Shinneman et al., 2013) and are generally intermixed in mesic areas at the same elevations as spruce/fir and lodgepole forests (Peet, 1978), which typically burn at high severity. Considering that failed fire lines in aspen stands were associated with the lowest amount of fire growth (Figure 5), this may suggest that fire growth decreases in the presence of aspen forests (i.e., aspen potentially slows the rate of spread). This is consistent with observations of fire behavior in the presence of aspen from the southwestern USA (Harris et al., 2025), which showed that as aspen cover increased from <10% to over 25%, average daily fire growth decreased from 1112 to 368 ha/day (Harris et al., 2025). Fire lines may still fail in aspen stands despite decreases in fire growth because aspen are not fire‐resistant and readily burn over to resprout post‐fire (Margolis & Farris, 2014; Mutch, 1970).

Fire lines were more likely to be constructed within shrublands or “other” land cover types compared to aspen forests. In contrast, shrublands, grasslands, and “other” land cover types were less likely to be with a fire footprint compared to aspen stands (likely due to the boundary of the study region). Constructing more fire lines near these lower elevation vegetation types makes sense given that much of the wildland urban interface (which has greater operational infrastructure) occurs at lower elevations and these fire lines are likely constructed with the intent of preventing damage to human structures.

### Probability of suppression operation success

When considering all fire events from 2019 to 2023, or only fires <40,500 ha (100,000 acres) during the same period, land cover type contributed little to no explanation of whether a fire line was likely to hold. This suggests that the traits of tree species—while undoubtedly important for their evolution in fire prone landscapes—have little effect on the success of suppression operations at this spatial and temporal scale, as predicted fire line success was not correlated with stand composition (Appendix S1: Figures S5 and S6). Instead, extreme conditions largely predict whether lines will hold or fail.

Fire growth was the most important predictor in our model that includes all fire events with suppression operations from 2019 to 2023. It is important to note that the 2020 and 2022 fire seasons accounted for some of the highest severity and largest fires recorded in this region (Coop et al., 2022; Higuera & Abatzoglou, 2021; Parks et al., 2025) and that extreme fire weather explains the explosive growth of wildfires during years of widespread burning (Balch et al., 2024; Coop et al., 2022). While this aligns with our prediction that fire growth mediated by extreme fire weather would be the primary predictor for the success of fire suppression operations, we observed lower fire growth associated with both failed and held fire lines in aspen stands (Figure 5). This may suggest that although fire growth is a good proxy for fire weather at landscape scales, there are interactions between fire weather and stand composition. Suppression bias also interacts with extreme climatic conditions, leading to fires that are difficult to contain (Kreider et al., 2024). By actively suppressing small fires that are easy to contain, fire suppression policies inadvertently create a filter that only large and difficult to manage fires are able to pass through (Kreider et al., 2024). This is evident from our use of two models, one that contains all fire events and another that excludes large fire events over 40,500 ha (100,000 acres). Without large fire events, most fire lines hold (18% failure rate; Table 1). However, when we include the four large fire events, the area burned increases over 6‐fold (71,000 vs. 444,000 ha) and we observe a doubling of held lines (1196 vs. 2813), and a 6‐fold increase in failed lines (258 vs. 1482). Combined, our results suggest that as climates change and we see more extreme atmospheric moisture deficits, paired with extreme wind conditions, we can expect increasing challenges to fire line effectiveness during suppression operations.

Spatial eigen vectors were also important predictors in both sets of models (Figure 3), with a high degree of variability that overlapped with many other predictor variables. This suggests that the success of fire lines was influenced by the spatial structure of our predictor variables (Povak et al., 2020). This is not surprising because fire, topography, and the distribution of species in the southern Rocky Mountains are influenced by space (Balch et al., 2024; Peet, 1978, 2000; Povak et al., 2020). Interestingly, slope was consistently positively correlated with predicted fire line success, such that fire lines were predicted to succeed on slopes above 12°. This appears counter to evidence that steeper slopes (>25°) have higher rates of fire spread (Butler et al., 2007), and to the difficulty associated with working on steeper slopes. A disproportionate number of held lines from a small number of fire events such as the Cow Creek or Sylvan fires which had 100% success on slopes above 25° could contribute to this trend (Appendix S1: Table S3). However, across all fires, few fire lines were constructed on steep slopes (over 25°) and accounted for only 4.6% of all held lines and 2.6% of all failed lines. There may be a selection bias in the data towards held lines on steeper slopes as it is unlikely that wildland firefighters construct fire lines on steeper slopes unless other topographic or environmental features promote confidence in lines holding (i.e., only building fire lines on steep slopes under the most ideal circumstances).

### Limitations

We only investigated the role of aspen on fire suppression operation success from 2019 to 2023 due to the temporal range of the remotely sensed map for aspen presence (Cook et al., 2024). High‐resolution remote sensing promoted a landscape scale understanding of the role of stand composition in fire line success. Landscape scales are often the scale at which policy is administered; however, using 10 × 10 m pixels does simplify the environmental, climatic, and topographic conditions that fires burn in. Fine‐scale variations in stand composition and structure (i.e., fuel), topography, and microclimate (such as wind or fuel moisture), play a role in the behavior of fire (Atchley et al., 2021; Hayes et al., 2024), which ultimately influences the success of fire suppression operations. For example, conifers regenerating below a mature aspen canopy are likely to increase fuel loads (Nesbit et al., 2023) but are unlikely to be captured by remote sensing methods. Further, some areas of the southern Rocky Mountains are impacted by increased fuel loads from fire suppression, such as lower elevation forests. These areas have had fuel reduction treatments to thin forest fuel loads, which may interact with the success of fire suppression operations (Moghaddas & Craggs, 2007) but was not explicitly explored. We did not include a model to investigate the trends in fire suppression operations for only our four largest fires because management decisions from individual fires would have an outsized impact on observed trends. Future investigations will benefit from incorporating many large fire events to determine how suppression operation success varies when directly compared to smaller fire events. Finally, suppression resources (e.g., aerial crafts) and tactics (e.g., back burning) likely influence fire line effectiveness, although these variables were not explicitly included. Future research targeted at understanding the environmental conditions and incident response resources at finer scales may explain more of the variation in fire suppression operation success in the future.

### Conclusions

Recognizing the patterns of fire line construction in forests and adjacent landscapes and the effectiveness of these lines based on stand composition is important for planning and implementing fire suppression operations. By using ICS‐209‐PLUS daily situation reports for fire events (St. Denis et al., 2023) and high‐resolution remotely sensed maps of the distribution of aspen canopy in the southern Rocky Mountains (Cook et al., 2024), we provide evidence that burned areas were less likely to be within aspen stands than stands of other tree species. Further, we show that fire lines were more likely to be constructed next to aspen than other tree species, but fire lines were less likely to be constructed next to aspen than shrublands or “other” land cover types. We did not see increased success of suppression operations adjacent to aspen stands. Instead, suppression operations were generally successful under less extreme burning conditions. Under anomalous burning conditions, extreme fire growth (spurred by atmospheric moisture deficits and wind) correlated with the 6‐fold increase in the failure of fire lines with fire growth over 3600 ha consistently correlated with failure of predicted fire lines. These results suggest that at the landscape‐scale in the southern Rocky Mountains extreme fire conditions override any potential buffering mechanisms of quaking aspen (e.g., decreased fire severity or spread; Harris et al., 2025) for fire suppression operations. Converting high elevation conifer forests to aspen near the wildland urban interface in the western United States would likely not affect fire suppression effectiveness under extreme growth days. As climates change and we see more extreme atmospheric moisture deficits, paired with extreme wind conditions, we can expect increasing challenges to wildfire suppression operations across the western United States.

## CONFLICT OF INTEREST STATEMENT

The authors declare no conflicts of interest.

## Supporting information


Appendix S1.

## Data Availability

Data on wildland fires and suppression operations are available publicly online in the National Interagency Fire Center Open Data Site (https://data-nifc.opendata.arcgis.com/) using the search term “Operational Data Archive YYYY” where YYYY represents individual years included in the study from 2019 to 2023. All code (Carter et al., 2026) is available in Zenodo at https://doi.org/10.5281/zenodo.18840103.
