# Supplementary material for: Fire lines adjacent to aspen are unlikely to hold during extreme burning conditions in southern Rocky Mountain forests
Source: Ecol Appl. 2026 May 8;36:e70249. doi: 10.1002/eap.70249 (PMC13155191; doi:10.1002/eap.70249)
Supplement: Supplementary file 1 — Appendix S1. [file EAP-36-e70249-s001.pdf]

Appendix S1

Fire lines adjacent to aspen are unlikely to hold during extreme burning conditions in southern Rocky Mountain forests

Trevor A. Carter, Jennifer K. Balch, Maxwell C. Cook, Sarah J. Hart

Ecological Applications

Table S1. The odds ratios of whether stands of specific composition were more or less associated with burned area as compared to aspen dominant stands and the odds ratio of whether stands of specific composition were more or less associated with fire lines as compared to aspen dominant stands. All tests were statistically significant ($p < 0.05$), the values in parentheses denote the range of the 95% confidence interval from a Fisher test.

	**Odds ratio of whether species will burn compared to aspen**	**Odds ratio of fire lines construction compared to aspen**
Douglas-Fir	4.05 (4.04 - 4.06)	0.572 (0.563 - 0.58)
Gambel Oak	3.46 (3.46 - 3.47)	0.652 (0.642 - 0.663)
Grassland	0.634 (0.633 - 0.635)	0.977 (0.96 - 0.994)
Lodgepole	5.3 (5.29 - 5.31)	0.486 (0.479 - 0.494)
Other	0.702 (0.701 - 0.704)	1.22 (1.2 - 1.24)
Pinyon/Juniper	1.68 (1.68 - 1.69)	0.753 (0.741 - 0.765)
Ponderosa	3.43 (3.42 - 3.44)	0.713 (0.702 - 0.724)
Spruce/Fir	2.94 (2.94 - 2.95)	0.613 (0.604 - 0.623)
Shrubland	0.71 (0.709 - 0.712)	1.19 (1.17 - 1.21)

Table S2. The stand area for major tree species or associations in hectares and percent of (1) the Southern Rocky Mountains + 10 km buffer, (2) burned area, and (3) the area adjacent to fire line construction.

Name	Southern Rockies (ha | %)	Burned (ha | %)	Adjacent to Fire Lines (ha | %)
Aspen	950,000 | 6.59	14,900 | 3.17	198 | 4.53
Douglas-Fir	1,110,000 | 7.66	67,000 | 14.3	512 | 11.7
Gambel Oak	653,000 | 4.53	34,100 | 7.27	297 | 6.81
Grassland	1,350,000 | 9.39	13,500 | 2.88	176 | 4.03
Lodgepole	1,230,000 | 8.53	95,700 | 20.4	623 | 14.3
Other	1,230,000 | 8.5	13,600 | 2.89	220 | 5.03
Pinyon/Juniper	1,570,000 | 10.9	41,000 | 8.72	411 | 9.42
Ponderosa	1,580,000 | 10.9	81,600 | 17.4	776 | 17.8
Spruce/Fir	1,640,000 | 11.4	73,500 | 15.6	602 | 13.8
Shrubland	3,120,000 | 21.6	34,900 | 7.42	550 | 12.6

Table S3. The total number of fire lines, number of fire lines on slopes greater than or equal to 12° (modelled threshold), and the number of fire lines on slopes greater than or equal to 25 (threshold from Butler et al. 2007) for both engaged held and engaged failed lines. The percentage below each count represents the percent of lines within each subset relative to the total number of engaged fire lines for each fire. Fire names in bold represent large fire events (n = 4) that were excluded in secondary analyses.

**Name**	**Total EH**	**EH ≥ 12°**	**EH ≥ 25°**	**Total EF**	**EF ≥ 12°**	**EF ≥ 25°**
403	101 (88.6%)	56 (49.1%)	5 (4.39%)	13 (11.4%)	12 (10.5%)	0 (0%)
Black Feather	7 (30.4%)	2 (8.7%)	0 (0%)	16 (69.6%)	3 (13%)	0 (0%)
Calwood	17 (89.5%)	11 (57.9%)	0 (0%)	2 (10.5%)	0 (0%)	0 (0%)
**Cameron Peak**	492 (83.7%)	155 (26.4%)	20 (3.4%)	96 (16.3%)	28 (4.76%)	2 (0.34%)
Cerro Pelado	47 (65.3%)	25 (34.7%)	1 (1.39%)	25 (34.7%)	10 (13.9%)	0 (0%)
Chris Mountain	13 (86.7%)	9 (60%)	6 (40%)	2 (13.3%)	2 (13.3%)	1 (6.67%)
Coalmine	13 (76.5%)	5 (29.4%)	0 (0%)	4 (23.5%)	3 (17.6%)	0 (0%)
Cow Creek	1 (100%)	1 (100%)	1 (100%)	0 (0%)	0 (0%)	0 (0%)
Decker	155 (83.3%)	111 (59.7%)	3 (1.61%)	31 (16.7%)	19 (10.2%)	4 (2.15%)
East Canyon	54 (98.2%)	46 (83.6%)	9 (16.4%)	1 (1.82%)	0 (0%)	0 (0%)
**East Troublesome**	106 (64.2%)	38 (23%)	1 (0.606%)	59 (35.8%)	26 (15.8%)	1 (0.606%)
El Valle	15 (100%)	8 (53.3%)	0 (0%)	0 (0%)	0 (0%)	0 (0%)
Grizzly Creek	187 (87.8%)	105 (49.3%)	25 (11.7%)	26 (12.2%)	21 (9.86%)	8 (3.76%)
**Hermit's Peak**	831 (50.5%)	175 (10.6%)	7 (0.425%)	816 (49.5%)	206 (12.5%)	6 (0.364%)
High Park	12 (70.6%)	2 (11.8%)	0 (0%)	5 (29.4%)	0 (0%)	0 (0%)
Hope	1 (100%)	0 (0%)	0 (0%)	0 (0%)	0 (0%)	0 (0%)
Lefthand	25 (89.3%)	20 (71.4%)	3 (10.7%)	3 (10.7%)	2 (7.14%)	0 (0%)
Little Mesa	0 (0%)	0 (0%)	0 (0%)	1 (100%)	0 (0%)	0 (0%)

Lowline	52 (92.9%)	23 (41.1%)	1 (1.79%)	4 (7.14%)	1 (1.79%)	1 (1.79%)
Middle Fork	15 (78.9%)	9 (47.4%)	7 (36.8%)	4 (21.1%)	3 (15.8%)	0 (0%)
Middle Mamm	8 (66.7%)	5 (41.7%)	1 (8.33%)	4 (33.3%)	4 (33.3%)	0 (0%)
Midnight	180 (67.2%)	33 (12.3%)	0 (0%)	88 (32.8%)	33 (12.3%)	0 (0%)
Monday Creek	6 (85.7%)	3 (42.9%)	0 (0%)	1 (14.3%)	1 (14.3%)	0 (0%)
Morgan Creek	20 (95.2%)	11 (52.4%)	0 (0%)	1 (4.76%)	1 (4.76%)	0 (0%)
**Mullen**	188 (42.6%)	19 (4.31%)	0 (0%)	253 (57.4%)	50 (11.3%)	10 (2.27%)
Plumtaw	41 (78.8%)	21 (40.4%)	0 (0%)	11 (21.2%)	4 (7.69%)	0 (0%)
Reveille	1 (100%)	0 (0%)	0 (0%)	0 (0%)	0 (0%)	0 (0%)
Saint Charles	70 (100%)	67 (95.7%)	11 (15.7%)	0 (0%)	0 (0%)	0 (0%)
Simms	4 (80%)	1 (20%)	0 (0%)	1 (20%)	0 (0%)	0 (0%)
Spring Creek	53 (94.6%)	41 (73.2%)	4 (7.14%)	3 (5.36%)	2 (3.57%)	0 (0%)
Sylvan	3 (100%)	3 (100%)	3 (100%)	0 (0%)	0 (0%)	0 (0%)
Thorpe	6 (100%)	0 (0%)	0 (0%)	0 (0%)	0 (0%)	0 (0%)
Titan	9 (81.8%)	1 (9.09%)	0 (0%)	2 (18.2%)	0 (0%)	0 (0%)
Trail Springs	7 (100%)	6 (85.7%)	0 (0%)	0 (0%)	0 (0%)	0 (0%)
Williams Fork	71 (87.7%)	41 (50.6%)	20 (24.7%)	10 (12.3%)	9 (11.1%)	6 (7.41%)
YMCA	2 (100%)	2 (100%)	0 (0%)	0 (0%)	0 (0%)	0 (0%)

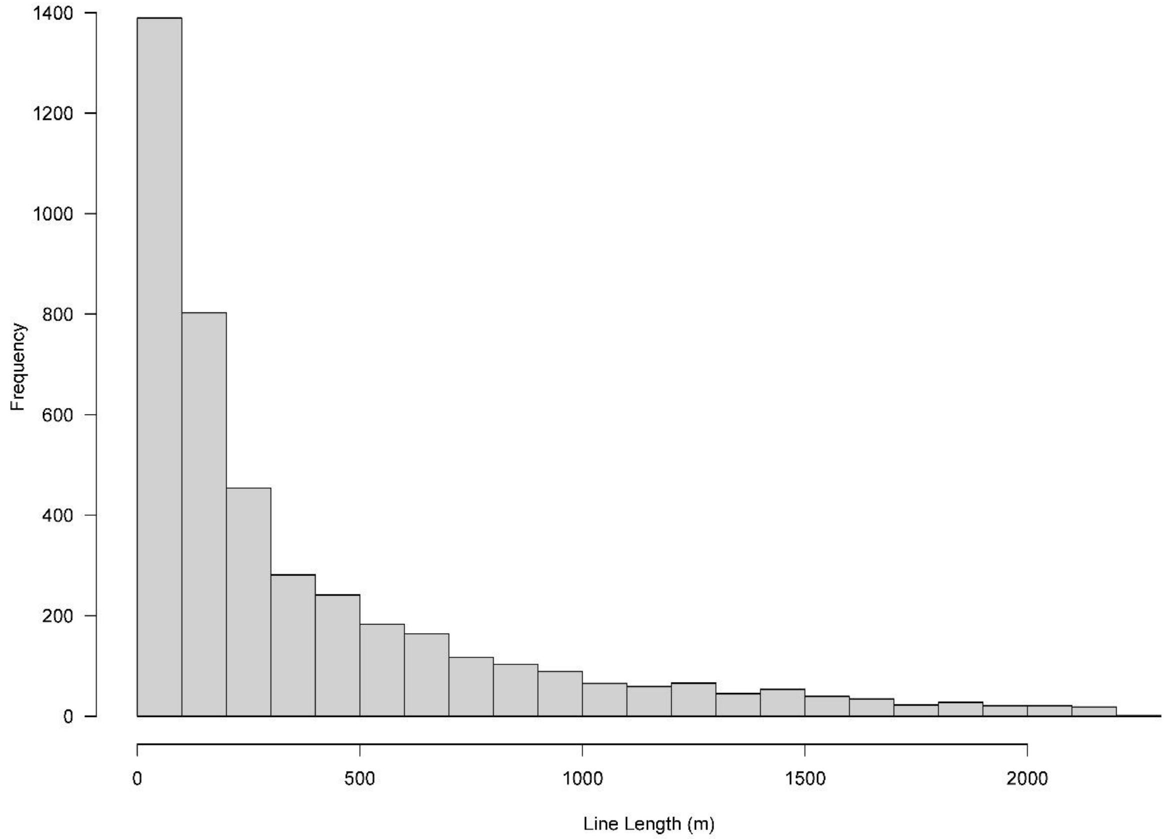

Figure S1. Distribution of line lengths both failed and held fire lines included in the study.

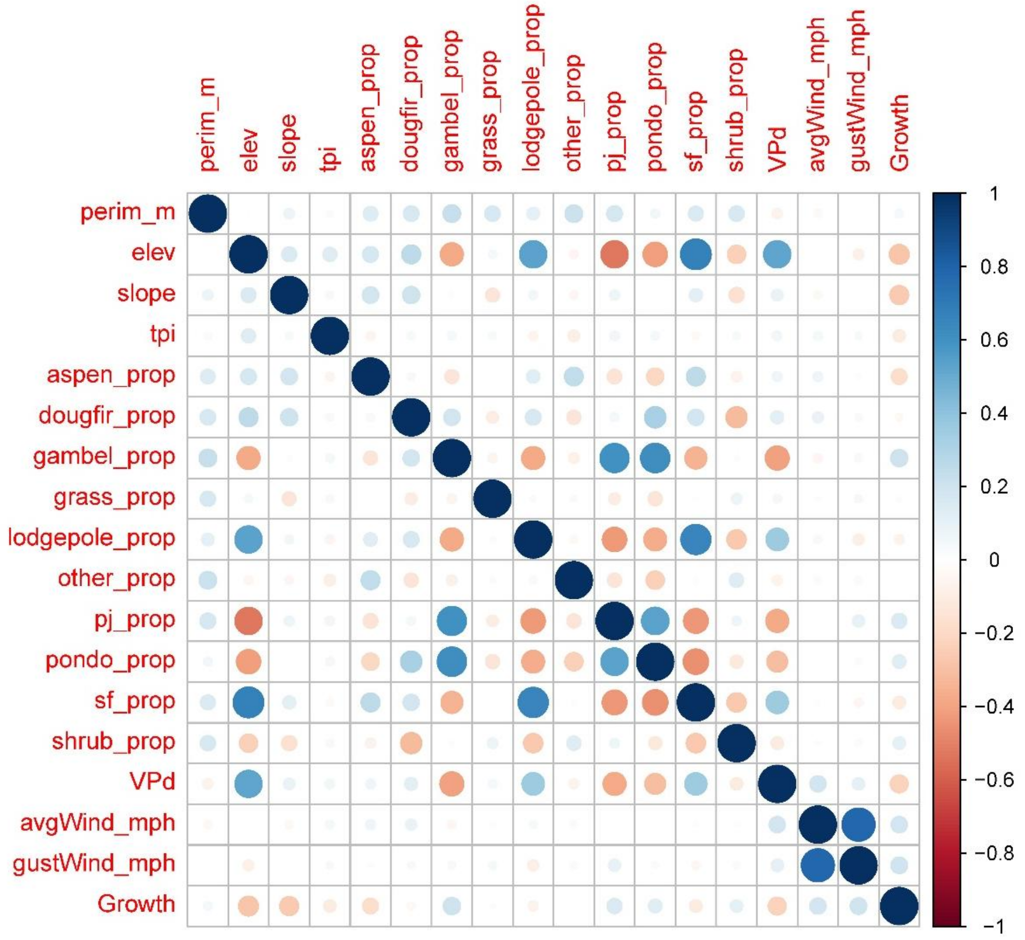

Figure S2. Pairwise correlation of predictor variables included in the random forest model that included all fire events (excluding spatial eigenvectors).

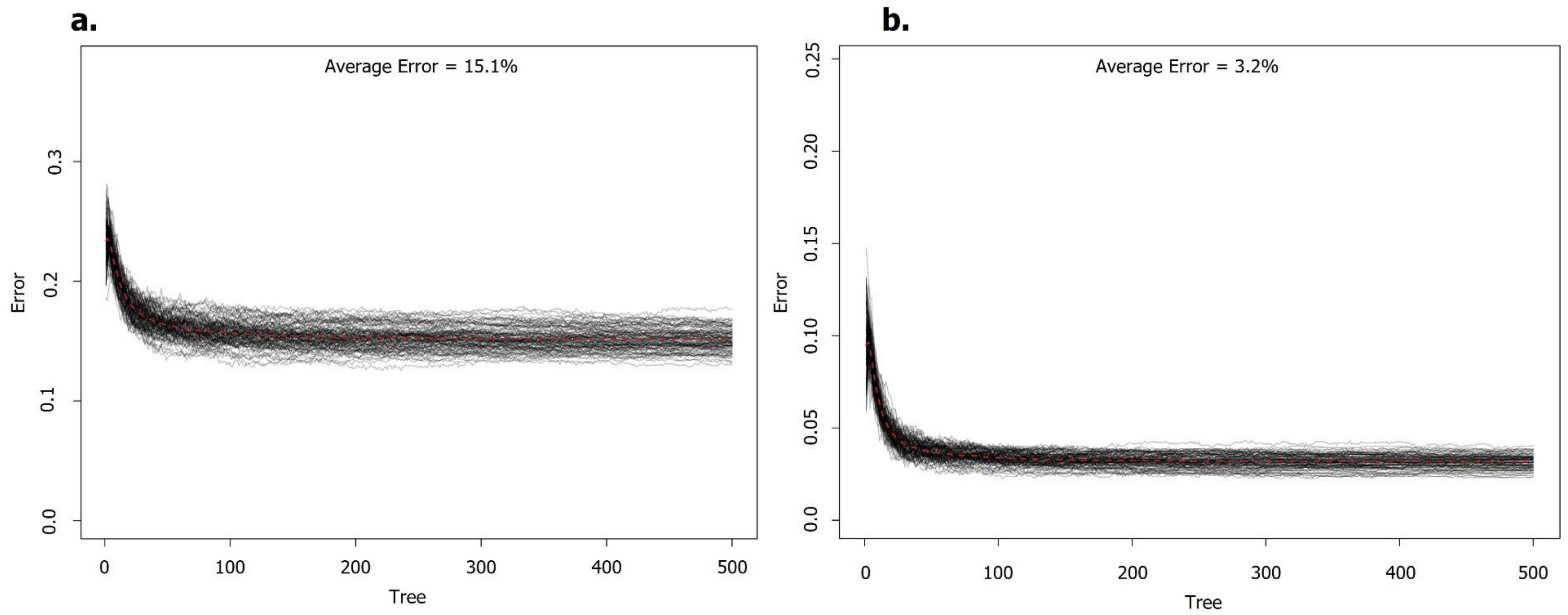

Figure S3. Out of bag error for each of the 100 model runs (black lines) for the random forest models that included (a) all fire events, and (b) excluded mega-fire events. The dashed red line represents the average error across all 100 models 15.1% and 3.2% respectively.

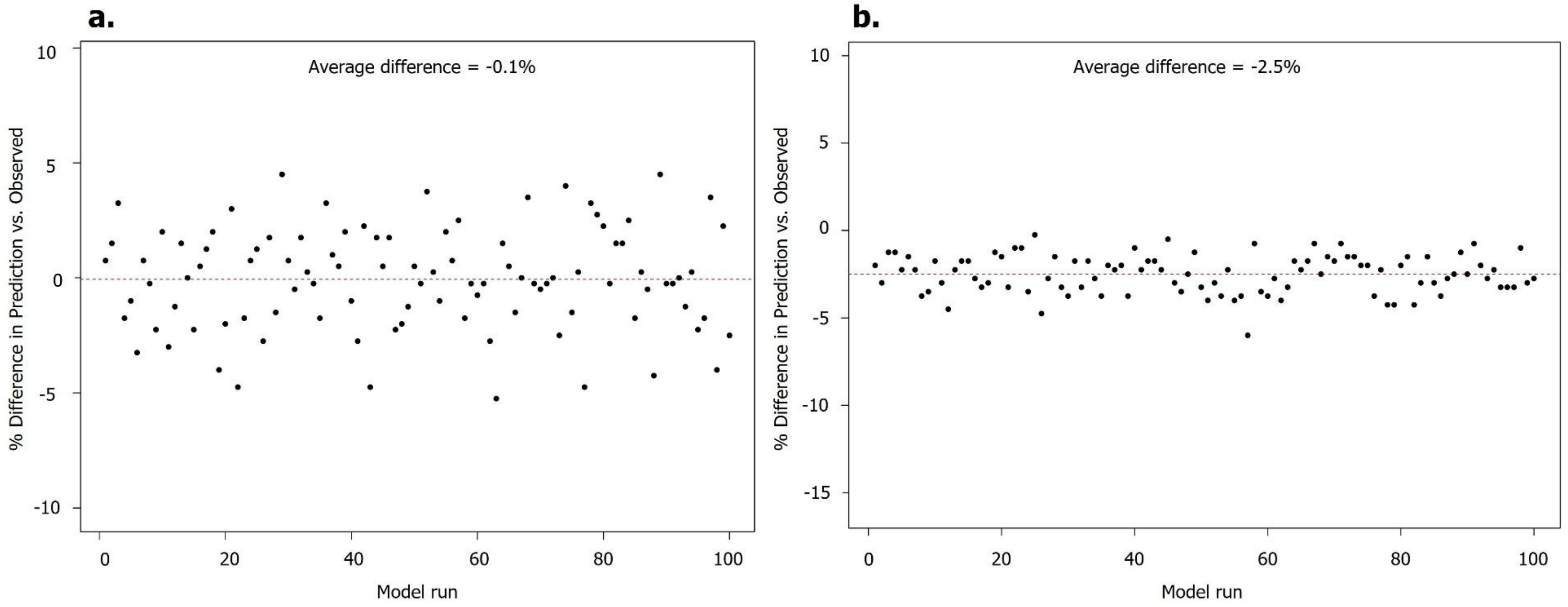

Figure S4. Points represent the average difference in predicted vs. observed for each of the n = 100 models for the models that include (a) all fire events, and (b) exclude mega-fires. The dashed red line represents the average difference in predicted vs. observed at -0.1% and -2.50% respectively.

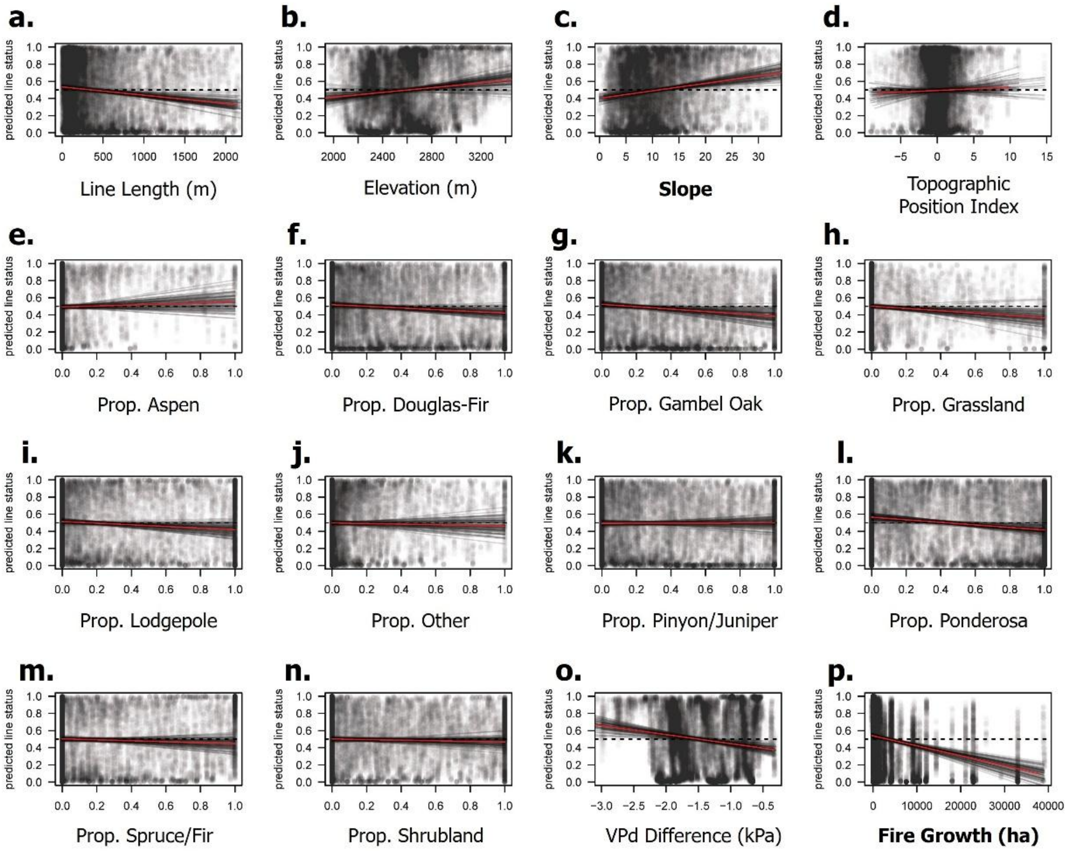

Figure S5. Partial effects of line length (a), elevation (b), slope (c), topographic position index (d), forest stand composition (e-n), deviation from 30 year normal for vapor pressure deficit (o), and fire growth (p) from the testing data on the predicted probability of fire lines holding (1 = predicted to succeed, 0 = predicted to fail). These random forest models and subsequent regression models include all fire events Each solid black line represents a single regression using the testing data and predicted line status from 1 of 100 random forest model runs. Points represent the distribution of the data. The red line represents the average partial effect across 100 runs. Dashed lines represent equal predicted probability of lines holding or failing. Panels with bold x-axis labels indicate which partial effects consistently correlate across all 100 model runs (i.e., all effects positive, or all effects negative).. These models explained on average 24.0% of the variation in predicted line status.

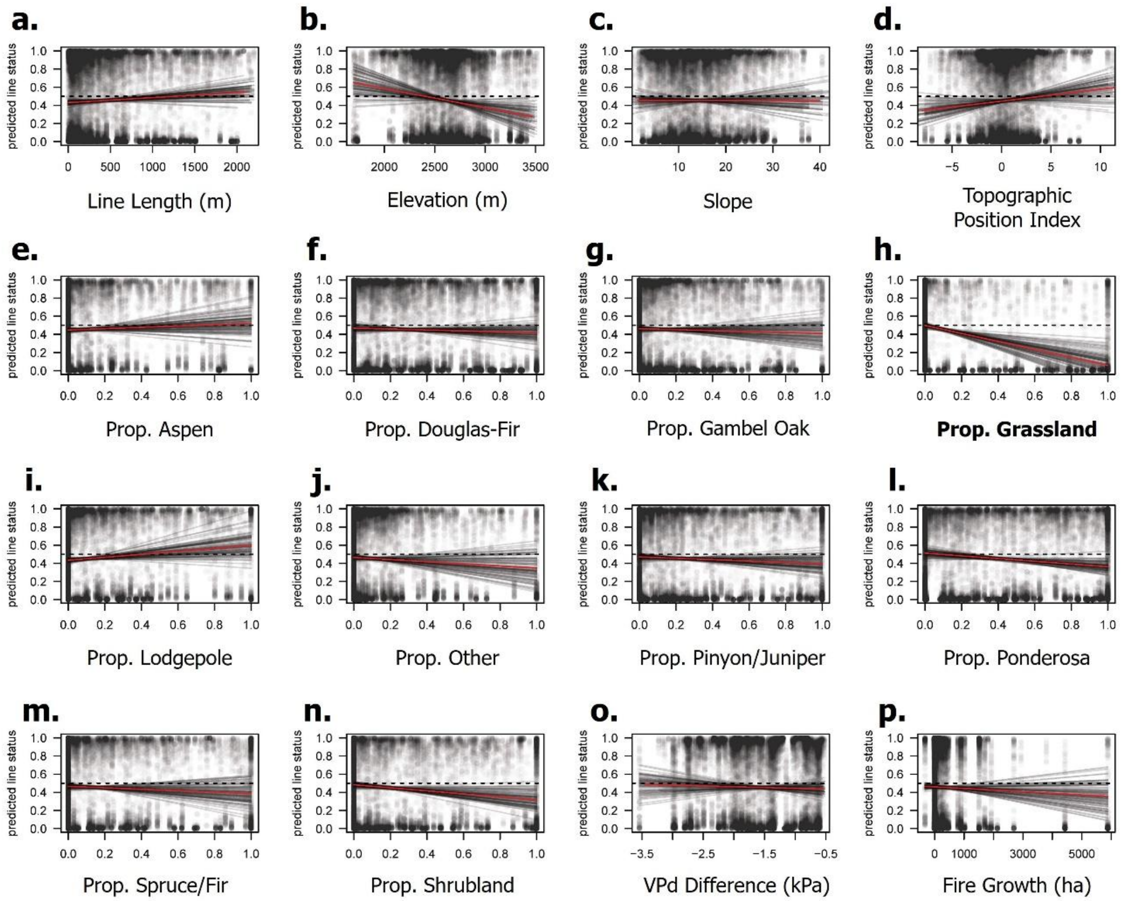

Figure S6. Partial effects of line length (a), elevation (b), slope (c), topographic position index (d), forest stand composition (e-n), deviation from 30 year normal for vapor pressure deficit (o), and fire growth (p) from the testing data on the predicted probability of fire lines holding (1 = predicted to succeed, 0 = predicted to fail). These random forest models and subsequent regression models excluded the four large fire events. Each black line represents a single regression using the testing data and predicted line status from 1 of 100 random forest model runs. Points represent the distribution of the data. The red line represents the average partial effect across 100 runs. Dashed lines represent equal predicted probability of lines holding or failing. Panels with bold x-axis labels indicate which partial effects were statistically significant ($p < 0.05$) for more than 5 of 100 model runs. These models explained on average 12.9% of the variation in predicted line status.

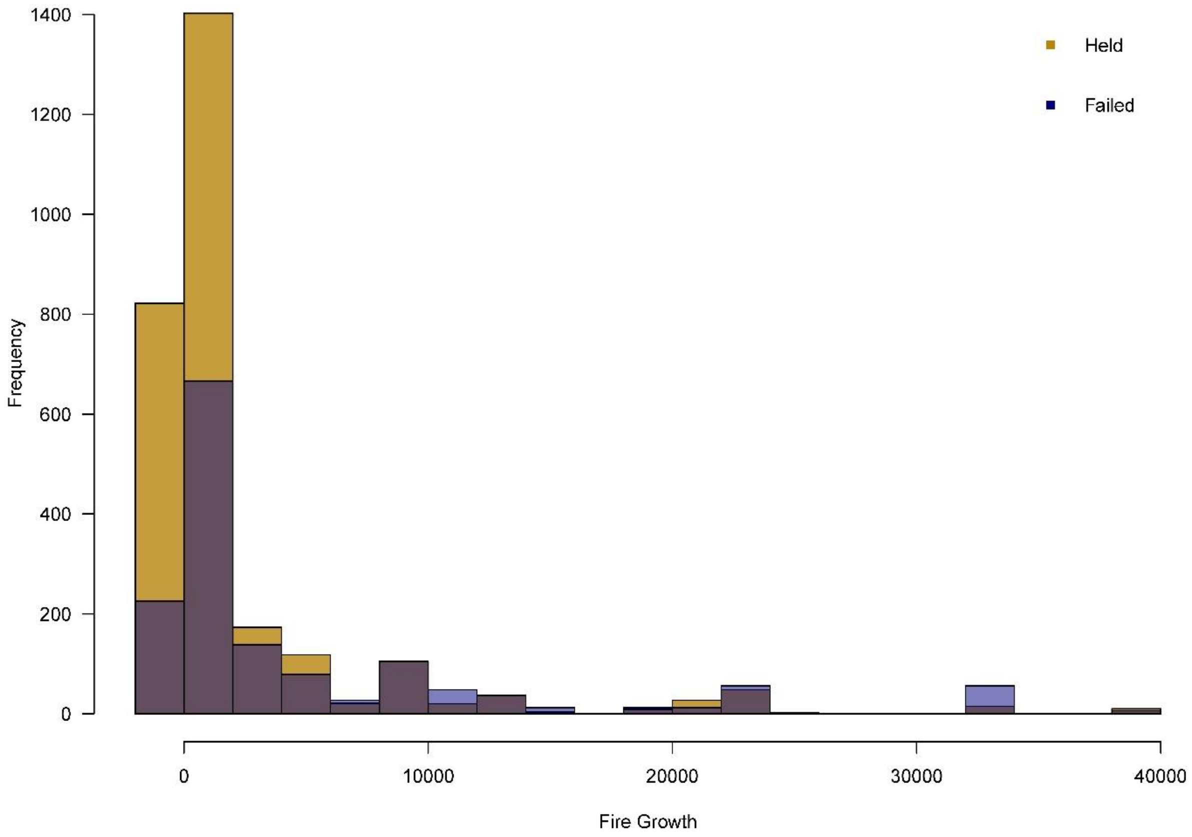

Figure S7. Distribution of fire growth for held (gold) and failed (navy) fire lines.

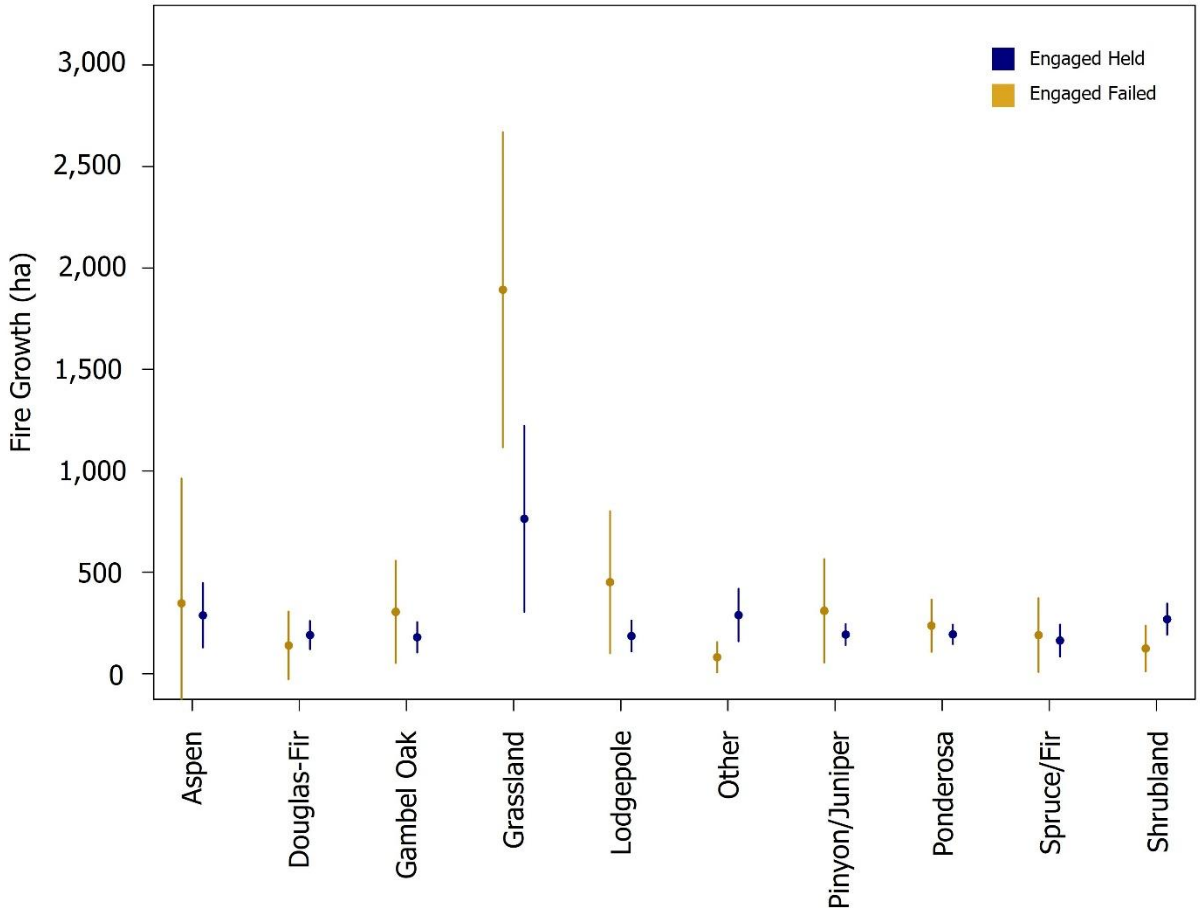

Figure S8. Fire growth across non-mega fire events associated with held (blue points and segments) and failed (yellow points and segments) fire lines based on stand composition. Points represent the average fire growth associated with each category (e.g., held lines in spruce/fir forests). Segments represent the 95% confidence interval around the mean using the formula for 95% confidence intervals assuming a Gaussian distribution.
